# Supplementary material for: Coral reef restoration efforts in Latin American countries and territories
Source: PLoS One. 2020 Aug 5;15(8):e0228477. doi: 10.1371/journal.pone.0228477 (PMC7406059; doi:10.1371/journal.pone.0228477)
Supplement: Attachment — Submitted filename: PONE-D-20-01163_reviewer_with comments.pdf [file pone.0228477.s003.pdf]

PLOS ONE

Coral reef restoration efforts in Latin American countries and territories

--Manuscript Draft--

**Manuscript Number:**	PONE-D-20-01163
**Article Type:**	Research Article
**Full Title:**	Coral reef restoration efforts in Latin American countries and territories
**Short Title:**	Coral reef restoration in Latin America
**Corresponding Author:**	Elisa Bayraktarov, PhD<br>University of Queensland<br>Brisbane, Queensland AUSTRALIA
**Keywords:**	coral reef restoration; Caribbean; Central America; Eastern Tropical Pacific; Latin America; Low-tech coral farming; Spanish-speaking countries; solutions to the global coral crisis
**Abstract:**	Coral reefs worldwide are degrading due to climate change, overfishing, pollution, coastal development, bleaching and diseases. In areas where natural recovery is negligible or protection through management interventions insufficient, active restoration becomes critical. The Reef Futures symposium in 2018 brought together over 400 reef restoration experts, businesses, and civil organizations, and galvanized them to save coral reefs through restoration or identify alternative solutions. The symposium highlighted that solutions and discoveries from long-term and ongoing coral reef restoration projects in Spanish-speaking countries in the Caribbean and Eastern Tropical Pacific were not well known internationally. Therefore, a meeting of scientists and practitioners working in these locations was held to compile the data on the extent of coral reef restoration efforts, advances and challenges. Here, we present unpublished data from 12 coral reef restoration case studies from five Latin American countries, describe their motivations and techniques used, and provide estimates on total annual project cost per unit area of reef intervened, spatial extent as well as project duration. We found that most projects used direct transplantation, the coral gardening method, micro-fragmentation or larval propagation, and aimed to optimize or scale-up restoration approaches (51%) or provide alternative, sustainable livelihood opportunities (15%) followed by promoting coral reef conservation stewardship and re-establishing a self-sustaining, functioning reef ecosystem (both 13%). Reasons for restoring coral reefs were mainly biotic and experimental (both 42%), followed by idealistic and pragmatic motivations (both 8%). The median annual total cost from all projects was $93,000 USD (range: $10,000 USD - $331,802 USD) (2018 dollars) and intervened a median spatial area of 1 ha (range: 0.06 ha - 8.39 ha). The median project duration was 3 years; however, projects have lasted up to 17 years. Project feasibility was high with a median of 0.7 (range: 0.5 - 0.8). This study closes the knowledge gap between academia and practitioners and overcomes the language barrier by providing the first comprehensive compilation of data from ongoing coral reef restoration efforts in Latin America.
**Order of Authors:**	Elisa Bayraktarov, PhD
	Anastazia T Banaszak
	Phanor Montoya Maya
	Joanie Kleypas
	Jesús E Arias-González
	Macarena Blanco
	Johanna Calle Triviño
	Nufar Charuvi
	Camilo Cortés Useche
	Victor M Galván

Powered by Editorial Manager® and ProduXion Manager® from Aries Systems Corporation

	Miguel A García Salgado
	Mariana Gnecco
	Sergio D Guendulain García
	Edwin A. Hernández Delgado
	José A Marín Moraga
	María Fernanda Maya
	Sandra Mendoza Quiroz
	Samantha Mercado Cervantes
	Megan Morikawa
	Gabriela Nava
	Valeria Pizarro
	Rita I Sellares-Blasco
	Samuel E Suleimán Ramos
	Tatiana Villalobos Cubero
	María Villalpando
	Sarah Frías-Torres
**Additional Information:**	
**Question**	**Response**
**Financial Disclosure**<br><br>Enter a financial disclosure statement that describes the sources of funding for the work included in this submission. Review the submission guidelines for detailed requirements. View published research articles from *PLOS ONE* for specific examples.<br><br>This statement is required for submission and **will appear in the published article** if the submission is accepted. Please make sure it is accurate.	The author(s) received no specific funding for this work.

**Unfunded studies**<br>Enter: *The author(s) received no specific funding for this work.*<br><br>**Funded studies**<br>Enter a statement with the following details:<br>• Initials of the authors who received each award<br>• Grant numbers awarded to each author<br>• The full name of each funder<br>• URL of each funder website<br>• Did the sponsors or funders play any role in the study design, data collection and analysis, decision to publish, or preparation of the manuscript?<br>• **NO** - Include this sentence at the end of your statement: *The funders had no role in study design, data collection and analysis, decision to publish, or preparation of the manuscript.*<br>• **YES** - Specify the role(s) played.<br><br>* typeset	
**Competing Interests**<br><br>Use the instructions below to enter a competing interest statement for this submission. On behalf of all authors, disclose any competing interests that could be perceived to bias this work—acknowledging all financial support and any other relevant financial or non-financial competing interests.<br><br>This statement **will appear in the published article** if the submission is accepted. Please make sure it is accurate. View published research articles from *PLOS ONE* for specific examples.	The authors have declared that no competing interests exist.

Powered by Editorial Manager® and ProduXion Manager® from Aries Systems Corporation

**NO authors have competing interests**<br><br>Enter: *The authors have declared that no competing interests exist.*<br><br>**Authors with competing interests**<br><br>Enter competing interest details beginning with this statement:<br><br>*I have read the journal's policy and the authors of this manuscript have the following competing interests: [insert competing interests here]*<br><br>* typeset	
**Ethics Statement**<br><br>Enter an ethics statement for this submission. This statement is required if the study involved:<br><br>• Human participants<br>• Human specimens or tissue<br>• Vertebrate animals or cephalopods<br>• Vertebrate embryos or tissues<br>• Field research<br><br>Write "N/A" if the submission does not require an ethics statement.<br><br>General guidance is provided below. Consult the submission guidelines for detailed instructions. **Make sure that all information entered here is included in the Methods section of the manuscript.**	N/A

Powered by Editorial Manager® and ProduXion Manager® from Aries Systems Corporation

**Format for specific study types**<br><br>**Human Subject Research (involving human participants and/or tissue)**<br>• Give the name of the institutional review board or ethics committee that approved the study<br>• Include the approval number and/or a statement indicating approval of this research<br>• Indicate the form of consent obtained (written/oral) or the reason that consent was not obtained (e.g. the data were analyzed anonymously)<br><br>**Animal Research (involving vertebrate animals, embryos or tissues)**<br>• Provide the name of the Institutional Animal Care and Use Committee (IACUC) or other relevant ethics board that reviewed the study protocol, and indicate whether they approved this research or granted a formal waiver of ethical approval<br>• Include an approval number if one was obtained<br>• If the study involved *non-human primates*, add *additional details* about animal welfare and steps taken to ameliorate suffering<br>• If anesthesia, euthanasia, or any kind of animal sacrifice is part of the study, include briefly which substances and/or methods were applied<br><br>**Field Research**<br><br>Include the following details if this study involves the collection of plant, animal, or other materials from a natural setting:<br>• Field permit number<br>• Name of the institution or relevant body that granted permission	
**Data Availability**<br><br>Authors are required to make all data underlying the findings described fully available, without restriction, and from the time of publication. PLOS allows rare exceptions to address legal and ethical concerns. See the PLOS Data Policy and FAQ for detailed information.	Yes - all data are fully available without restriction

A Data Availability Statement describing where the data can be found is required at submission. Your answers to this question constitute the Data Availability Statement and **will be published in the article**, if accepted.<br><br>**Important:** Stating 'data available on request from the author' is not sufficient. If your data are only available upon request, select 'No' for the first question and explain your exceptional situation in the text box.<br><br>Do the authors confirm that all data underlying the findings described in their manuscript are fully available without restriction?	
**Describe where the data may be found in full sentences. If you are copying our sample text, replace any instances of XXX with the appropriate details.**<br><br>• If the data are **held or will be held in a public repository**, include URLs, accession numbers or DOIs. If this information will only be available after acceptance, indicate this by ticking the box below. For example: *All XXX files are available from the XXX database (accession number(s) XXX, XXX.).*<br>• If the data are all contained **within the manuscript and/or Supporting Information files**, enter the following: *All relevant data are within the manuscript and its Supporting Information files.*<br>• If neither of these applies but you are able to provide **details of access elsewhere**, with or without limitations, please do so. For example:<br><br>*Data cannot be shared publicly because of [XXX]. Data are available from the XXX Institutional Data Access / Ethics Committee (contact via XXX) for researchers who meet the criteria for access to confidential data.*<br><br>*The data underlying the results presented in the study are available from (include the name of the third party*	The data is provided within the submitted supplementary information

and contact information or URL).<br>• This text is appropriate if the data are owned by a third party and authors do not have permission to share the data.<br><br>* typeset	
Additional data availability information:	

Manuscript Click here to access/download;Manuscript;Coral restoration manuscript revised_nofigs.docx

Coral reef restoration efforts in Latin American countries and territories

Elisa Bayraktarov[1]*, Anastazia T. Banaszak[2], Phanor Montoya Maya[3], Joanie Kleypas[4], Jesús E. Arias-González[5], Macarena Blanco[6], Johanna Calle Triviño[5,6,15], Nufar Charuvi[7], Camilo Cortés Useche[5,15], Victor Galván[8], Miguel A. García Salgado[9], Mariana Gnecco[3], Sergio D. Guendulain García[2], Edwin A. Hernández Delgado[10,11,12], José A. Marín Moraga[4], María Fernanda Maya[3], Sandra Mendoza Quiroz[2,13], Samantha Mercado Cervantes[8], Megan Morikawa[6], Gabriela Nava[9], Valeria Pizarro[14], Rita I. Sellares-Blasco[15], Samuel E. Suleimán Ramos[10], Tatiana Villalobos Cubero[4], María Villalpando[15], Sarah Frías-Torres[16,17]

[1] Centre for Biodiversity and Conservation Science, The University of Queensland, Brisbane, Queensland, Australia
[2] Investigación Integral para la Conservación de Arrecifes, Universidad Nacional Autónoma de México (UNAM), Puerto Morelos, Quintana Roo, Mexico
[3] Corales de Paz, Cali, Colombia
[4] Raising Coral Costa Rica, Costa Rica
[5] Departamento de Recursos del Mar, Centro de Investigación y de Estudios Avanzados del Instituto Politécnico Nacional, Mérida, Yucatán, Mexico
[6] Iberostar Hotels & Resorts, *Wave of Change*, Dominican Republic, Mexico
[7] Fundación Calipso, Taganga, Magdalena, Colombia
[8] Fundación Grupo Puntacana (FGPC), Punta Cana, La Altagracia Province, Dominican Republic
[9] Oceanus A.C., Chetumal, Quintana Roo, Mexico
[10] Sociedad Ambiente Marino (SAM), San Juan, Puerto Rico
[11] University of Puerto Rico, Department of Environmental Sciences, San Juan, Puerto Rico
[12] University of Puerto Rico, Centre for Applied Tropical Ecology and Conservation, San Juan, Puerto Rico
[13] SECORE International, Inc., Hilliard, Ohio, USA
[14] Fundación para la Investigación y Conservación Biológica Marina ECOMARES, Cali, Valle del Cauca, Colombia
[15] Fundación Dominicana de Estudios Marinos (FUNDEMAR), Santo Domingo, Dominican Republic
[16] Vulcan, Seattle, Washington, USA
[17] Smithsonian Institution, Washington, District of Columbia, USA

* Corresponding author

Email address: e.bayraktarov@uq.edu.au

Abstract

Coral reefs worldwide are degrading due to climate change, overfishing, pollution, coastal development, bleaching and diseases. In areas where natural recovery is negligible or protection through management interventions insufficient, active restoration becomes critical. The Reef Futures symposium in 2018 brought together over 400 reef restoration experts, businesses, and civil organizations, and galvanized them to save coral reefs through restoration or identify alternative solutions. The symposium highlighted that solutions and discoveries from long-term and ongoing coral reef restoration projects in Spanish-speaking countries in the Caribbean and Eastern Tropical Pacific were not well known internationally. Therefore, a meeting of scientists and practitioners working in these locations was held to compile the data on the extent of coral reef restoration efforts, advances and challenges. Here, we present unpublished data from 12 coral reef restoration case studies from five Latin American countries, describe their motivations and techniques used, and provide estimates on total annual project cost per unit area of reef intervened, spatial extent as well as project duration. We found that most projects used direct transplantation, the coral gardening method, micro-fragmentation or larval propagation, and aimed to optimize or scale-up restoration approaches (51%) or provide alternative, sustainable livelihood opportunities (15%) followed by promoting coral reef conservation stewardship and re-establishing a self-sustaining, functioning reef ecosystem (both 13%). Reasons for restoring coral reefs were mainly biotic and experimental (both 42%), followed by idealistic and pragmatic motivations (both 8%). The median annual total cost from all projects was $93,000 USD (range: $10,000 USD - $331,802 USD) (2018 dollars) and intervened a median spatial area of 1 ha (range: 0.06 ha - 8.39 ha). The median project duration was 3 years; however, projects have lasted up to 17 years. Project feasibility was high with a median of 0.7 (range: 0.5 - 0.8). This study closes the knowledge gap between academia and practitioners and overcomes the language barrier by providing the first comprehensive compilation of data from ongoing coral reef restoration efforts in Latin America.

Introduction

Active restoration, the process of assisting the recovery of an ecosystem that has been degraded, damaged, or destroyed [1], may be increasingly necessary on coral reefs, once it has been determined that the natural recovery of corals is hindered [2]. The goal of any restoration action is to eventually establish self-sustaining, sexually reproducing populations with enough genetic variation enabling them to adapt to a changing environment [3-5].

Coral reef restoration may play a particularly important role where coral species are threatened with extinction. The Caribbean Elkhorn coral, *Acropora palmata*, and Staghorn coral, *A. cervicornis*, were once widely distributed and among the major reef-building species in the region [6]. Both species are now listed as Critically Endangered on the International Union for Conservation of Nature (IUCN) Red List [7] as a result of major losses in cover of both species throughout the Caribbean since the 1970s [8]. Management programmes have not aided in the recovery of *A. palmata* [9]. In this context, active restoration of these species is essential to recover their ecosystem functions in the Caribbean region.

Several techniques are used for the restoration of coral reefs. The most common techniques are based on asexual methods such as direct transplantation, coral gardening, and micro-fragmentation [10]. An alternative technique, larval propagation, is based on the collection of gametes and the consequent culturing of embryos and larvae, after which the coral spat are either grown in *ex situ* aquaria to larger-sized colonies or are outplanted onto degraded reefs at approximately one month old [11]. While the techniques used to restore coral reefs are reviewed elsewhere (e.g. [10, 12-14]), here we focus on direct transplantation, coral gardening, micro-fragmentation, and larval propagation as the techniques most-commonly employed by the case studies in the study area. One of the oldest techniques used in coral reef restoration is direct transplantation of corals [15], which involves the harvesting of coral colonies from a donor site and their immediate transplantation to a restoration site or re-attaching colonies that have been dislodged by a ship grounding, storm or hurricane [16]. The coral gardening approach was developed to scale-up restoration while reducing the stress on

donor colonies. Fragments of corals are harvested from donor colonies, grown in nurseries to a threshold size [17] before being transplanted onto a degraded reef [18, 19]. Nurseries can be ocean-based (*in situ*) or land-based (*ex situ*). *In situ* nurseries are typically located at well-lit sites safe from predation, storm surges, and wave energy, and are regularly maintained and cleaned by physical removal of algal growth [20]. However, strategic siting of ocean nurseries can promote the recruitment of fish assemblages that eat biofouling, thus may significantly reduce person-hours spent in nursery cleaning [21]. *In situ* nurseries can have many shapes and sizes. For example, they can consist of floating mid-water structures built using ropes, mesh or cages [21-24], structures placed on concrete, tables or frames [25], PVC 'trees' [26], PVC grids or dead coral bommies [27]. *Ex situ* nurseries typically use flow-through large aquaria or raceways, and require continuous access to electricity, water quality monitoring, and control of temperature and light availability [28]. Micro-fragmentation is an approach especially useful for slow-growing massive corals. This technique involves the fragmentation of parts of a massive coral donor to yield multiple ~1 cm^2 fragments. The fragments are placed close to each other on either artificial substrates or on the surface of dead coral colonies. The micro-fragments, as they recognize neighbouring fragments as kin, grow towards each other and fuse [29]. Ideally, they are outplanted to the degraded reef at a size of ~6 cm^2 [29, 30]. Larval propagation involves the breeding of corals from eggs and sperm. Studies describing this technique typically report the use of raceways with seawater flow-through systems where coral spawn is collected from the wild, fertilization is assisted, embryos are cultured to larvae, which are settled onto substrates and then transported and seeded onto a degraded coral reef [31-33]. This process has also been referred to as larval enhancement, sexual propagation, sexual coral cultivation or larval reseeding [12]. As an emerging larval propagation technique, larval restoration concentrates coral larvae over enhancement plots on the degraded reef to facilitate coral larvae settlement directly to the substrate, without the need of laboratory facilities [34]. The main advantages of the larval propagation techniques are that they increase the genetic diversity among restored coral populations thus enabling increased rates of adaptation and improved resilience in the context of climate change

[35], and they have the potential to be used over large scales while reducing the cost [31]. Also, they do not cause damage to the parent colonies.

While efforts in the USA, Australia or places where European scientists conduct their research are well described in the published literature and disseminated at conferences, there is a paucity of documentation on coral reef restoration projects carried out by practitioners in the Caribbean and Eastern Tropical Pacific. Reasons for this lack of exchange may be the language barrier, lack of interest in knowledge transfer between higher and lower income countries or cultural differences as well as lack of funding. In 2018, the Reef Futures symposium was held in the Florida Keys, USA and attended by over 400 delegates. The aim of this international meeting was to 'bring together experts from around the world to share the latest science and techniques for coral reef restoration while kicking off a global effort to dramatically scale-up the impact and reach of restoration as a major tool for coral reef conservation and management'. The conference was organized by the Coral Restoration Consortium, which is 'a community of practice comprised of scientists, managers, coral restoration practitioners, and educators dedicated to enabling coral reef ecosystems to survive the 21st century and beyond' [36]. Within the Reef Futures conference, we convened a meeting of scientists and practitioners involved in active coral reef restoration in the Latin- and Centro-American Caribbean as well as the Eastern Tropical Pacific to fill the knowledge gap between academia and practitioners in the region and overcome the language barriers in coral reef restoration. Here, we showcase the advances and share the lessons learned from 12 restoration case studies from the Caribbean and Eastern Tropical Pacific. We provide a comprehensive compilation of unpublished data from coral reef restoration efforts where we outline the techniques that were employed, the motivations and objectives of each project, total project cost per unit area per year, spatial extent of intervention, and project duration. This work provides the most complete data set on total project cost and feasibility of coral reef restoration from practical cases that may guide decisions required to establish new restoration projects in the future.

Approach

Data collection

The co-authors of this work contributed data and descriptions of their restoration projects which constitute the case studies used here. The coral reef restoration projects were carried out in Latin American countries and territories in the Caribbean and Eastern Tropical Pacific (**Fig. 1**). The data obtained included estimates on total annual project cost, spatial extent of area intervened, project duration, and an estimate on the project reaching specific objectives within a fixed period of time. The motivations for each restoration project were adopted from [10, 37, 38] and classified as biotic, experimental, idealistic, legislative, and pragmatic (**Table 1**).

Table 1: Five motivation categories for carrying out coral reef restoration projects and examples.

Motivation category	Examples
Biotic	Biodiversity enhancement (e.g., native species, habitat creation, ecosystem connectivity, ecological resilience)
Experimental	Improve restoration approaches, technologies, and methods. Answer ecologically-based research questions
Idealistic	Cultural reasons (e.g., recreation, tourism, medicinal/ceremonial substances, spiritual importance, aesthetic value)
	Social reasons (e.g., community involvement, job creation, nature education, environmental outreach)
	Political reasons (e.g., raising environmental profile)
Legislative	Restoration after environmental impact (e.g., ship-grounding, mining, oil spill, hurricane damage)
	Biodiversity offset (e.g., threatened species, threatened ecological communities)
Pragmatic	Enhance ecosystem services (e.g., fisheries production)
	Enhance ecosystem services (e.g., water quality improvement, pollution prevention)
	Enhance ecosystem services (e.g., coastal protection, erosion control, bank stabilisation)
	Enhance ecosystem services (e.g., carbon sequestration, carbon offsets)

The objectives of coral reef restoration projects can be highly diverse and dependent on the specific project as well as its location. In this study, the restoration practitioners were asked to provide the objectives for their restoration projects, which were specific, measurable, achievable, repeatable and time-bound (SMART; [3]). We modified the six primary objectives observed by Hein et al. [39] into the following categories: 1) enhance ecosystem services for the future; 2) optimize/scale-up restoration approaches; 3) promote coral reef conservation stewardship; 4) provide alternative, sustainable livelihood opportunities; 5) reduce coral population declines and ecosystem degradation; and 6) re-establish a self-sustaining, functioning reef ecosystem.

The total estimated project cost includes both capital and operating costs. Capital costs are those used for planning, land acquisition, construction, and financing [40]. These may also include costs for laboratory/infrastructure, boats and dive equipment. Operating costs are those used for maintenance, monitoring, equipment repair and replacement [40] and may include salaries, housing for scientific/implementation teams, air for SCUBA tanks, gasoline for boat engines, and replacement of computers. Coral reef practitioners were asked to estimate the total cost for restoration interventions based on the guidelines for standardised reporting of costs for management interventions for biodiversity conservation [41] and are provided as United States Dollars (USD) per hectare of coral reef intervened per year in 2018 USD.

The project spatial extent is the coral reef area intervened by the restoration project and is reported in hectares. Spatial extent is not provided for each project since not all restoration case studies have an objective to increase the area of restored habitat. For instance, some projects are aimed at developing new restoration techniques, using coral nurseries as a tool to stimulate public awareness and engagement, for educational purposes, or as a tourist attraction.

The project duration is the time during which the restoration project has existed until the present, or the time during which the restoration cost was budgeted for and is provided in years. All projects described here are ongoing and active throughout 2019.

The feasibility is the likelihood that each specific project objective can be reached successfully with the interventions at hand and within the outlined project duration. It is ideally measured as the likelihood of success in returning the ecosystem function and resilience of an ecosystem through restoration [42]. This overall restoration project feasibility is rarely reported in the published literature because a standardised method to measure restoration success is largely missing [40]. Here, restoration practitioners estimated the feasibility of the restoration interventions they employed to achieve their specific project objectives. Feasibility is given as a ratio between 0 and 1 and can be interpreted as the likelihood of success to reach a specific objective within the duration of the restoration project. Practitioners provided a minimum, maximum and the best guess for the project feasibility.

Results

Data from a total of 12 coral reef restoration projects carried out by practitioners in the Spanish-speaking Caribbean and Eastern Tropical Pacific were compiled and are summarised in **Table 2**. The supplementary material contains more detailed information about each restoration case study. Information was gathered from Colombia (Alianza Coralina Taganga, Corales de Paz, and ECOMARES), Costa Rica (Raising Coral Costa Rica), the Dominican Republic (FUNDEMAR, the Iberostar Group, and Fundación Grupo Puntacana), Mexico (Oceanus A.C., CORALIUM at Universidad Nacional Autónoma de México, and the Iberostar & CINVESTAV Group), and Puerto Rico (Sociedad Ambiente Marino) (**Figure 1**). Note that the Fundación Grupo Puntacana has two restoration programs of which one is focused on coral gardening (Program 1) and one is directed towards micro-fragmentation (Program 2). These were treated as independent projects for analytical purposes. The restoration projects use techniques that include direct transplantation, coral gardening, micro-fragmentation, and larval propagation (**Figure 2;** Supplementary information **Table S1**).

Table 2: Summary of the 12 restoration projects in the Caribbean and Eastern Tropical Pacific. Cost values are given in 2018 USD. More detailed information can be found in the supplementary material. Abbreviations: Fundación Dominicana de Estudios Marinos, Inc. (FUNDEMAR), Fundación Grupo Puntacana (FGPC), and Sociedad Ambiente Marino (SAM).

Country, Location, Organization	Technique employed (type of nursery)	Targeted coral species	Motivations	Specific project objectives	Strategy for outplanting	Spatial extent of project	Estimated project budget and funding bodies/partners	Estimated project feasibility
				**Implemented and in progress as of 2019**				
Colombia, Taganga, Caribbean Sea, Alianza Coralina Taganga	A floating mid-water nursery built of PVC tubes is located 5 m below the water surface and anchored to the sandy bottom at 13 m depth. Coral fragments are produced by micro-fragmentation of donor colonies, which are then attached to cement cookies and outplanted once they reach a diameter of 7 cm. Each cement cookie is connected via a plastic screw to a mesh frame in the coral nursery with a carrying capacity of 50 – 80 cookies per frame. Corals of opportunity are presently used.	*Montastraea cavernosa; Porites porites; Millepora* sp.	The primary motivation is idealistic following social reasons such as community education and engagement. The secondary motivation is experimental i.e., to improve management and develop standardized restoration protocols.	1) to develop a training centre for the sustainable use of marine resources and ecological restoration; 2) to establish a community-based coral reef monitoring system for Taganga Bay and coral nurseries therein with the possible expansion of monitoring to other areas; 3) to develop a management plan for Taganga Bay as a marine reserve, which is governed by the local community; and 4) to create a financed organization, which aims to facilitate long-term ecological reef restoration and research in Taganga Bay	Corals grown in the mid-water nursery will be outplanted by drilling holes in the natural substrate with a pneumatic drill and inserting the plastic nails of the cement cookies carrying the coral fragments into the holes (supported by epoxy glue where necessary). All outplanted corals at the restoration site will be monitored at least once per month while they reattach to the natural substrate.	The spatial extent of the project is currently a matter of negotiation that depends on the capacity to recruit coral gardeners from the local community and to obtain a permit to carry out the ecological restoration work in Taganga Bay.	An estimated budget for the project is $500,000 USD over the next two years. Forty percent of this budget is self-funded by local stakeholders to accelerate the capacity of coral growth and maintenance of coral outplanting through local capacity building. A large proportion of the estimated project budget will be directed to activities such as education, community engagement and training while a minor part will be focused on growing and outplanting corals to the restoration site. Members from the local community are trained as coral gardeners to identify corals of opportunity, carry out coral micro-fragmentation and maintain the nursery.	best guess = 0.5; minimum = 0.2; maximum = 0.9
Colombia, San Andres and Providencia Islands, Caribbean Sea, Corales de Paz	The project employs the coral gardening technique. Rope nurseries are floating at 4 to 6 m below the water surface (**Fig. 2a**). Micro-fragmentation is also	*Acropora palmata*; *A. cervicornis*; *Porites porites*; *Madracis decactis*; *Pseudodiploria*	The primary project motivation is biotic i.e., to enhance coral reef biodiversity, while the secondary motivation is pragmatic i.e., to enhance the ecosystem	1) to generate an annual stock of > 5,000 coral fragments from four reef-building species per island; 2) to transplant 5,000 coral colonies per ha^{-1} yr^{-1} per island from year two of the project, for a total of 30,000 coral transplants over six	Nursery-grown corals as well as micro-fragments will be outplanted to the reef using a unique mix of marine cement and a colloid adjuvant to improve fluidity and	The total spatial extent is six hectares (area of outplant) by year four distributed over three hectares at each of the two islands.	The total estimated budget is $900,000 USD resulting in an annual expenditure of $37,500 USD ha^{-1} yr^{-1}. Partners include the Secretary of Agriculture and Fisheries from the Government of the Archipelago of San Andrés,	best guess = 0.6; minimum = 0.5; maximum = 0.9

	being employed during the outplanting phase.	*clivosa; P. astreoides; Millepora complanata*	services of fisheries, tourism and coastal protection by the local coral reefs.	hectares in three years; 3) to achieve a 25% increase in selected coral reef health indicators (i.e., live coral cover, coral settlement, fish biomass, and rugosity) at the intervened sites; 4) to design and implement an effective system of protection and restoration of intervened reef areas that encourages conservation and contributes to the sustainability of benefits derived from these reefs together with relevant social actors; and 5) to quantify the ecosystem services of intervened reef areas in current and future scenarios of intervention, variability and climate change	reduce runoff. Outplanting density will be 5,000 individuals per ha of reef.		Providencia and Santa Catalina, the provincial environmental authority CORALINA, Conservation International Colombia, and the NGO Corales de Paz. Both islands are within the Seaflower Biosphere Reserve in the Colombian Caribbean. The first phase of the project was financed by all participating organizations with support from MasBosques and BanCO2.	
Costa Rica, Golfo Dulce, Eastern Tropical Pacific, Raising Coral Costa Rica	The project employs the techniques coral gardening and micro-fragmentation. Coral fragments are often collected as fragments of opportunity and are grown in tree and rope nurseries, after which they are outplanted to the restoration site (**Fig. 2f**).	*Pocillopora* sp.; *Porites evermanni*; *P. lobate*; *Pavona gigantea*; *Pavona frondifera*; *Psammocora* sp.	The primary motivation of the project is experimental with the rationale to improve coral propagation techniques for growing corals in the Eastern Tropical Pacific, with an emphasis on answering questions of ecological concern. The secondary motivation is biotic i.e., to enhance biodiversity, ecosystem connectivity, and ecological resilience.	1) to define the best coral propagation and restoration techniques; 2) to establish a coral restoration program in Costa Rica; 3) to facilitate coral reef research to improve restoration work; and 4) to integrate local communities into coral restoration projects	Branching corals grown in the nursery are outplanted onto the substrate with cable ties attached to large nails. Future endeavours will attach corals grown on ropes in rope-nurseries directly to the substrate without separating them from the ropes. Massive and encrusting corals, which are grown on ceramic plugs, are outplanted by drilling holes into the substrate and inserting the stem of the plug with a small amount of marine epoxy or cement.	The project aims to restore 10 reef patches of 200-500 m^2 each within the next three years equalling a maximum intervened area of 0.5 ha.	The total project cost over the last 2.5 years was $120,000 USD. If in kind support (such as accommodation, university technical support, volunteer time, etc.) is included, these costs would be 100% higher, i.e., a total of $240,000 USD. The annual project budget was $35,000 USD for 2018, which was mostly composed of salaries ($15,000 USD) and logistics such as travel and boat rental ($15,000 USD). The remaining $5,000 were needed for material and consumables. The project is mainly financed by private donations and Raising Coral Costa Rica is currently initiating a fundraising campaign call to restore several thousand corals for Costa Rica and to scale-up coral propagation and restoration efforts.	best guess = 0.8; minimum = 0.6; maximum = 0.9
Dominican Republic,	The techniques coral gardening (rope and steel	*Acropora cervicornis*;	The primary motivation of the project is biotic	1) to propagate coral tissue of the endangered *A. cervicornis* using	Corals grown in eight underwater nurseries	FUNDEMAR's restoration project	FUNDEMAR is a largely self-sustainable organization that has	best guess = 0.7; minimum = 0.4;

Bayahibe, Caribbean Sea, FUNDEMAR	rod nurseries (**Fig. 2b**) and larval propagation (seeding coral recruits after cultivation using *in situ* SECORE Int.-designed floating pools (**Fig. 2c**)) and *ex situ* in a wet lab) are used to restore local coral populations.	*A. palmata; Colpophyllia natans; Diploria labyrinthiformis*; *Orbicella annularis*; *O. faveolata*;	with the rationale of biodiversity enhancement. The secondary motivation is legislative focused on restoration after environmental impact and as a biodiversity offset. However, the project has also idealistic motivations for cultural, social and political reasons.	the genetically diverse coral nurseries; 2) to enhance coral populations' genetic diversity and resilience to environmental changes by outplanting substrates (either SECORE´s cement or ceramic substrates or FUNDEMAR's cement "cookies") with settled coral recruits.	(holding more than 3 km of tissue) are outplanted with nails, cable ties and epoxy glue techniques. FUNDEMAR has already carried out coral outplanting at 12 restoration sites (**Table S3**, supplementary material). The project is monitoring two spawning sites used to deliver the spawning stocks for rearing coral larvae in an *ex situ* facility (**Table S2**, supplementary material). Corals reared by larval propagation settle on hand-made cement or ceramic substrates which are nailed or seeded on the reef.	aims to intervene on at least 0.5 hectares of degraded coral reef per year on a restoration schedule of one coral colony per square meter, transplanting around 2,000 *A. cervicornis* coral fragments of around 20-30 cm in diameter and seeding 2,000 recruit substrates (from 3-5 different species).	developed strategic alliances with private and public national and international institutions and with financial support for implementation of new projects. The total project cost per year is around $51,800 USD which includes costs for maintenance, staff salaries, boats, and keeping up the facility but excludes in-kind support from volunteers. Part of this funding ($18,400 USD per year) comes from the local, national and international alliances that FUNDEMAR has established. The coral reef restoration project has been financed by two grants and alliances with other organizations carrying out coral reef restoration.	maximum = 0.9
Dominican Republic, Bayahibe, Caribbean Sea, The Iberostar Group	The Iberostar Group uses the coral gardening technique to restore coral reefs and is currently in charge of two coral nurseries at two locations in the Dominican Republic. One of the nurseries is an *in situ* nursery and the other one is an *in situ* nursery connected to a land-based facility for research and to evaluate the genetic diversity of corals in the nursery.	*Acropora cervicornis; A. palmata; Diploria labyrinthiformis; Porites porites; P. astreoides; Orbicella annularis; O. faveolata; Agaricia agaricites*	Reef restoration in one of the main pillars of the *Wave of Change* movement initiated in 2018 by the international hotel chain Iberostar. *Wave of Change* aims at contributing to the conservation of the oceans by engaging with the tourism sector. The main motivations of this project are experimental, biotic and idealistic.	1) to determine current intraspecific diversity; 2) to enhance intra- and inter-specific diversity; 3) to maintain *in situ* and *ex situ* genetic bank; 4) to engage hotel clients and staff; 5) to characterise individual physiological traits of corals; 6) to enhance resilience in restored reefs	Despite the nursery still being developed, fragments of *A. cervicornis, P. porites* and *A. agaricites* have been maintained with 100% survival rate for three months to date.	No information on the spatial extent of area intervened is available yet, because the transplantation strategy is currently being developed. As part of this strategy, the group aims to identify coral genotypes potentially less susceptible to environmental stress.	The estimated budget spent from the beginning of the project in May 2018 to March 2019 is $100,000 USD, including materials and construction of the land-based facility and salaries. In 2018 alone, $40,000 USD were spent on construction (excluding salaries). The project is privately financed with annual funds destined to the *Wave of Change* initiative. From 2016 onwards, the group has taken over the responsibility of an *in situ* coral nursery that was set up and maintained in collaboration with FUNDEMAR. Research will be carried out by the scientific team of *Wave of Change* as well as by collaborating with international	best guess = 0.5; minimum = 0.2; maximum = 0.8

							scientists who can use the facility to conduct their studies. The interspecific diversity of nursery-grown corals is being addressed through genetic analyses in collaboration with the University of California at Santa Barbara. Finally, the facility will be used as an outreach centre to teach and raise awareness about topics such as coral biology, the importance of reefs, threats to marine ecosystems, etc. to hotel clients and staff.	
Dominican Republic, Punta Cana, Caribbean Sea, Fundación Grupo Puntacana (Program 1)	Nursery fragments are grown on A-Frames, tables and ropes at water depths between 3.5 and 5 m.	*Acropora. cervicornis*; *A. palmata*; *Orbicella* spp.; *Porites* spp.; *Pseudodiploria* spp.	The primary motivation of the project program is biotic and is focused on the enhancement of biodiversity and resilience. The secondary motivation is idealistic and concentrates on social reasons (e.g. development of alternative income opportunities for local communities and improved user experience for tourism, fisheries, ecosystem services etc.).	1) to prevent a potential local or regional disappearance of coral species through enhancement of successful sexual reproduction using fast growing, genetically diverse, nursery-reared fragments; 2) to reduce local environmental problems such as marine pollution, unsustainable wastewater treatment, uncontrolled fisheries and tourist carrying capacity; 3) to train local community members such as fishermen or dive centre staff in the installation and maintenance of coral nurseries and outplanting of nursery-grown corals; 4) to replicate the lessons learned in other parts of the Dominican Republic and other Caribbean island nations to improve coral reef restoration in Punta Cana; 5) to generate alternative income opportunities for members of the local community, especially for local fishermen	Nursery fragments are outplanted on the local, patchy, fringing reef using cable ties and galvanized nails at similar depths to fragments growing in the nursery.	Since 2014, a total of 8,810 *A. cervicornis* fragments (representing 5,394 linear meters of coral tissue) have been transplanted over almost 0.44 ha of degraded reef. Sexual reproduction has been consistently observed at both the nursery and surrounding outplanted sites.	The total estimated budget for 2018 was around $93,000 USD resulting in $211,363 USD ha^{-1} yr^{-1} when extrapolated from the actual area intervened (0.44 ha). This budget includes salaries, material, equipment, consumables, fixed-assets, infrastructure upkeep, and project-related expenses. For the next 3 years (2019 – 2021), if grant proposals submitted are approved, there is a plan to scale-up coral reef restoration efforts. These include an increase in the number of *A. cervicornis* fragments outplanted to approximately 5,000 fragments per year. FGPC estimates that over the next 3 years about 15,000 fragments can be transplanted over one ha of natural coral reef. The total estimated budget for the time interval 2019 – 2021 will be approximately $950,000 USD, thus equalling the total cost of $313,500 USD ha^{-1} yr^{-1}. The coral reef restoration programs are supported by the general budget of FGPC. Additional support is provided by private donations,	best guess = 0.8; minimum = 0.5; maximum = 0.9

							national and international grants and institutions such as Deutsche Gesellschaft für Internationale Zusammenarbeit (GIZ), The Nature Conservancy (TNC), Counterpart International (CPI), Caribbean Hotel and Tourism Association, Global Giving, and InterAmerican Development Bank (IDB) among others.	
Dominican Republic, Punta Cana, Caribbean Sea, Fundación Grupo Puntacana (Program 2)	The donor colonies (fragments of opportunity) are cut by a diamond band saw into approximately one cm^2 pieces, which are then attached to cement discs made in-house and deposited into flow-through raceways. This program consists of three phases. The first phase identifies the best conditions for high survival and fast growth of the micro-fragments in the *ex situ* nursery and develops the protocols for the approach. The second phase identifies adequate restoration sites and develops outplanting protocols. During this phase, methods, tools and equipment are tested. The third phase will scale-up outplanting efforts with micro-fragments.	*Pseudodiploria strigosa; P. clivosa; Porites astreoides; P. furcata; Orbicella annularis; Montastraea cavernosa*	The primary motivation of this project program is biotic (biodiversity enhancement), while the secondary is experimental (improve restoration approach, technology and methods). A tertiary motivation is idealistic (environmental education and outreach for the local community and tourists).	As above	Micro-fragments will be outplanted using established protocols.	By the end of the third phase, an estimate of 5,000 micro-fragments will be outplanted annually covering up to 200 m^2 per year.	The total budget for 2018 was around $30,000 USD. The project duration is three years and the total estimated budget is $850,000 USD (pending grant approvals). Funding bodies/partners are as above.	best guess = 0.6; minimum = 0.4; maximum = 0.9
Mexico, Chetumal, Caribbean Sea, Oceanus A.C.	This project uses the coral gardening approach where corals are grown in *in situ* nurseries. Fragments of opportunity	*Acropora palmata, A. cervicornis*; *A. prolifera*; *Porites* spp.; *Agaricia*	The primary motivation of this program is pragmatic i.e., to recover reef ecosystem health and promote	1) to promote the rehabilitation of coral reefs through transplantation of 10,000 colonies every year at different sites in the Gulf of Mexico and the Mexican	The restoration sites are selected according to a set of established criteria. Every new site requires between three	The spatial extent of total area intervened for all restoration activities since 2014 is	The total project budget was estimated to average $150,000 USD per year since 2014 to outplant 10,000 colonies every year with an outplanting schedule	best guess = 0.8; minimum = 0.5; maximum = 0.9

are rescued from donor areas and grown in the nurseries. Small concrete bases are attached to the reef and then corals from the nurseries are fixed to these structures (**Fig. 2d**). To increase the diversity at the restoration sites and promote natural resilience to climate change and local stressors, the program identifies the genetic material (genotypes) from healthy donor populations using the microsatellite technique. At least five genotypes are combined at each restoration site.	spp.; *Orbicella* spp.; *Diploria* spp.	recovery of environmental services of the reef as well as associated species populations and biomass with special emphasis on recovering protected and no-take areas. The secondary motivation is legislative, i.e., to restore coral reefs after environmental impacts such as ship-grounding or hurricanes depending on the location and site.	Caribbean; 2) to strengthen the resilience and adaptation potential of coral reefs by increasing diversity on restoration sites through the identification of genetic material from healthy donor populations that could be naturally resilient to climate change and local stressors; 3) to secure community and reef managers' engagement to build local restoration groups that work based on a self-sustainable strategy to multiply efforts, increasing benefits to local communities in the short and mid-term as well as helping the activities of the program to be maintained for a longer term	and five years of work until colonies of the first and second generation have grown to reproduce sexually. Every year, monitoring is carried out before and after transplantation at each of the sites to evaluate the survival and growth of restored corals. The overall average of transplant survival has been about 80%. At the oldest restoration sites initiated from 2013 onwards and maintained by the program, the outplanted coral fragments, which initially had average sizes of between 7 and 10 cm, have now (in 2019) grown to an average size of 30 cm in diameter. Some outplants have reached a diameter of up to 110 cm (**Fig. 2e**). About 30% of the transplants evaluated in 2019 at all sites had a size of 20 cm in diameter on average indicating that they have reached a reproductive size.	estimated as 6.3 ha to date.	of one coral colony per square meter. Therefore, the annual budget was estimated at $150,000 USD ha^{-1} yr^{-1}. The restoration work of Oceanus A.C. is mainly artisanal and requires intensive maintenance to achieve results. Therefore, restoration efforts can only be sustained if the local community is involved to guarantee restoration success. The restoration program has initiated the formation of local restoration groups mainly consisting of members of the local fishing communities and other local organizations as well as the private sector (e.g., hotels) to support the restoration efforts. The program also seeks to engage local communities, service providers such as diving shops, hoteliers and managers to build local restoration groups and form a restoration network that helps increase restoration efforts along the Mesoamerican Reef. Establishing this network and applying different restoration strategies depending on the local stakeholder involved is envisioned to allow the program to become self-sustainable in the long term. The main partners of Oceanus A.C. for the development and scaling-up of the program have been the Comisión Nacional de Áreas Naturales Protegidas (CONANP), Summit Foundation, the Mesoamerican Reef Fund, Fairmont Mayakobá and OHL Group, with local partners such as Acuario de Veracruz, Fundación de Parques y Museos de Cozumel, hotels from Playa del Carmen

							(Mayakobá chain) and from Mahahual and Xcalak, the Xcalak community, and tourist services providers from Cozumel, Puerto Morelos and Veracruz.	
Mexico, Mexican Caribbean, Caribbean Sea, CORALIUM, Universidad Nacional Autónoma de México	Since 2007, CORALIUM has been studying the basic biology of coral reproduction with the production of sexual recruits for restoration efforts beginning in 2011. Subsequently, it has focused on the development of low-cost techniques to scale-up the production of coral sexual recruits. This involves gamete collection in the wild, assisted fertilization and embryo husbandry in *ex situ* aquaria followed by outplanting of the sexual recruits to degraded reef sites. Restoration trials involve outplanting sexual recruits produced annually in the laboratory since 2011. From 2011 to 2014, the recruits were grown to juvenile size (up to 10 cm maximum diameter) in *ex situ* aquaria located in the Xcaret Ecopark. These colonies are now sexually mature as evidenced by the production of gametes in 2019. Since 2014, the research, in collaboration with SECORE International, has focused on scaling-up production and reducing costs by	*Acropora palmata; Orbicella faveolata; O. annularis; Diploria labyrinthiformis; Pseudodiploria strigosa*	This project undertakes science-based research to promote and scale-up best practices for coral restoration using sexual recruits (experimental motivation) and to increase genetic diversity in restoration efforts in the face of global climate change (biotic motivation).	1) to reduce costs of techniques using larval propagation of corals 100-fold; 2) to conduct research to improve survivorship of sexual recruits 20-fold; 3) to scale-up coral restoration techniques to ecologically significant scales over a 10-year period	To reduce costs, coral larvae are settled onto the artificial substrates and outplanted two weeks post-settlement (one-polyp stage). The substrates are placed manually into natural gaps formed by the reef framework without using cement or resin. New substrate designs are in the process of being tested to increase recruit survival from 0.1% at one-year post-settlement currently to a target of 10% and to improve substrate retention in the reef framework.	CORALIUM, in collaboration with SECORE International and Experiencias XCARET Aquarium have outplanted coral sexual recruits with sizes ranging from one polyp to colonies with an estimated volume of 500 cm^3 on eight degraded reefs along the Mexican Caribbean from Cancun to Xcalak (**Table S7**, supplementary material). In total, the area of outplants corresponds to 0.15 hectares.	The costs for the production and outplanting of sexual recruits between 2014 and 2018 is estimated at $15,000 USD per year and equals $100,000 USD ha^{-1} yr^{-1}. CORALIUM's research and restoration efforts have been funded by Universidad Nacional Autónoma de México, Comisión Nacional de Áreas Naturales Protegidas, Consejo Nacional de Ciencia y Tecnología, Comisión Nacional para el Conocimiento y Uso de la Biodiversidad, Alianza World Wildlife Fund – Fundación Carlos Slim, SECORE International, The Nature Conservancy and Experiencias XCARET. Through communication and outreach, CORALIUM has promoted the message that coral reef conservation efforts and adaptive protected areas management are key to any coral restoration efforts. In collaboration with SECORE International and The Nature Conservancy, CORALIUM has implemented an annual coral reproduction course focused at students, coral restoration practitioners and other stakeholders.	best guess = 0.7; minimum = 0.6; maximum = 0.9

	outplanting sexual recruits at the one polyp stage settled onto tetrapod-shaped substrates, designed by SECORE International.							
Puerto Rico, Culebra Island, Caribbean Sea, Sociedad Ambiente Marino	Coral nurseries were established in year 2003 and operated continuously for 15 years until the devastating impacts by hurricanes Irma and María in 2017. Following the hurricanes, tree coral nurseries were established at a depth of 9 m. Additional tree nurseries were established at a depth of 6-8 m, and at a depth of 7-12 m, to prevent further damage from coral bleaching and storm swells. Micro-fragmentation methods and direct coral cuttings have also been employed since the recent expansion. Direct transplantation has been conducted for emergency outplanting of fragments and/or detached colonies generated by vessel groundings, winter swells or hurricanes.	*A. cervicornis; A. palmata; A. prolifera; Dendrogyra cylindrus; O. annularis; O. faveolata; Madracis aurentenra; Porites divaricata; Eusmilia fastigiata*	The primary motivation of the project is biotic (i.e., to enhance biodiversity, coral reef connectivity and ecosystem resilience). The secondary motivation is experimental (i.e., testing alternative methods and designs, with aims to answer ecological research questions). The tertiary motivation is pragmatic (i.e., enhance the ecosystem services by improving shallow-water essential fish habitat, restoring depleted fisheries, restoring hurricane mechanical impacts, recovering shallow reef accretion following the 2005 post-bleaching mass coral mortality event; enhancing carbon sequestration, tourism, and coastal protection of local coral reefs). Also, an important local motivation is to restore coral reef ecological functions within areas formerly impacted by military training	1) to expand the annual stock in the nurseries of *A. cervicornis* to 8,000 colonies, of *A. palmata* to 2,500 colonies, *D. cylindrus* to 500 colonies, and *O. annularis* to 500 colonies; 2) to restore approximately 3 ha of degraded reef per year till 2022; 3) to outplant a minimum of 20,000 colonies of four species grown in the nurseries by year 2022, including 13,300 colonies of *A. cervicornis*, 5,000 colonies of *A. palmata,* 1,200 colonies of *D. cylindrus*, and 500 colonies of *O. annularis*; 4) to achieve a 25% increase in selected coral reef health indicators (i.e., live coral cover, fish biomass, and rugosity) at intervened sites for *A. cervicornis* and *A. palmata*; 5) to design and implement an effective community-based plan for the rehabilitation of intervened reef areas, which encourages conservation and rehabilitation of ecosystem functions, and to contribute to the sustainability of the benefits of coral reefs; 6) to quantify the ecosystem services of intervened reef areas in current and future scenarios of intervention, variability and climate change	Overall, in the time span of 2003-2017 approximately 60,000 coral colonies (mostly *A. cervicornis*) were harvested and outplanted to coral reefs in Culebra Island. Nursery-grown corals, fragments of opportunity of multiple species, as well as micro-fragments and cuttings are directly outplanted to the reef using Portland marine cement mixed with lime to neutralize pH. Cable ties and masonry nails are also used in the case of *A. cervicornis*. An outplanting schedule with a density of one individual per square meter of reef for *A. cervicornis* and of one colony per four square meters for other species is often followed.	The project has intervened an area of ca. 6 ha, but many of these corals were lost during the 2017 hurricanes. The projected spatial extent of reef rehabilitation by year 2023 in total will be 8.4 ha, with a potential to increase the area intervened depending on funding and on community-based volunteer support.	The funds projected towards restoration for the period of 2019 to 2023 are $1,327,206 (2018 USD), resulting in $158,189 USD per restored ha per year or an estimated investment of $50.26 USD per coral colony. These figures are based on the direct funds spent without accounting for in-kind contributions from the community. The real total estimated budget (including community-based in-kind support) for the period of 2019 to 2022 is $2,311,280 (2018 USD) resulting in a total annual expenditure of $275,480 (2018 USD) ha^{-1} or a total estimated expense of $87.53 per coral colony. Coral nurseries have been historically managed by SAM, and since 2011, in direct collaboration with the Centre for Applied Tropical Ecology and Conservation (CATEC) of the University of Puerto Rico – Río Piedras Campus, under a memorandum of agreement with the Puerto Rico Department of Natural and Environmental Resources (PRDNER), and NOAA Restoration Centers (NOAA-RC). The first 4-year sub-project of the post-hurricane long-term phase of the project will be financed through multiple sources, as described above. It will also involve extensive volunteer work, through a combination of	best guess = 0.7; minimum = 0.5; maximum = 0.9

			activities. Finally, the project is motivated by an idealistic rationale due to cultural reasons (i.e., community-based aim to restore formerly bombarded grounds by the U.S. Navy which used local coral reefs in Culebra Island to support naval training activities between 1901 and 1975, rescue and stewardship of local coral reefs) and due to social reasons (i.e., fostering increased community involvement, job creation, nature education, environmental outreach, hands-on training in coral farming and reef rehabilitation methods). More recently, the project is being motivated by legislative reasons (i.e., restoration of *A. palmata* and *A. cervicornis* as part of mitigatory compensation project).				strategies involving students, fishermen, NGOs, and an internship program. SAM also plans to involve the hospitality sector. There will also be a large focus on a combination of outreach, educational and hands on strategies to prepare the next generation of coral farmers and coral reef restoration researchers in Puerto Rico.	
				**Planned work**				
Colombia, Gorgona National Natural Park, Eastern Tropical Pacific, ECOMARES	The project is presently gathering scientific information for future needs on coral restoration in the area.	*Pocillopora damicornis; Pavona clavus*	The project is motivated by experimental reasons to improve restoration approaches for their use at Gorgona National Natural Park once restoration efforts are necessary.	1) to determine the feasibility of coral nurseries in the area; 2) to determine the minimum coral fragment size of *P. damicornis* for successful survival and growth in coral nurseries; 3) to find the optimal fragment size for outplanting in terms of survival	The group's expertise in outplanting has been focused in the most abundant coral species in the area, *P. damicornis*. For this coral species, Portland cement mixed with sand	N/A	The cost for running the projects have been lower than expected because they are mostly experimental and have not carried out formal coral reef restoration activities yet. In 2018 the budget was $10,000 USD. Partners: Universidad del Valle, Universidad	best guess = 0.7; minimum = 0.5; maximum = 0.9

				and coral growth; 4) to determine the effect of fish predation on *P. damicornis* during the outplanting; 5) to evaluate the use of enriched substrates for the massive coral species *P. clavus*	and freshwater was used. So far, no information is available to determine the spatial extent (area) of restored habitat that will be obtained.		Javeriana de Cali, and Gorgona National Natural Park.	
Mexico, Cozumel National Natural Park, Caribbean Sea, The Iberostar & CINVESTAV Group:	The project aims to start with the development of four genotyped coral nurseries, two for *Acropora palmata* (3 and 5 m water depth) and two for *A. cervicornis* (10 and 13 m water depth). Each nursery will have 5 structures with a carrying capacity of approximately 40 fragments each enabling growth of 800 corals at a time.	*Acropora palmata; A. cervicornis; Pseudodiploria* spp.; *Siderastrea* spp.; *Diploria labyrinthiformis; Orbicella* spp.	Coral reef restoration envisioned by both groups is mainly motivated by experimental, biotic (i.e., enhance biodiversity, ecosystem connectivity, and ecological resilience), idealistic and pragmatic reasons (i.e., enhanced water quality and ecosystem services, shallow-water essential fish habitat, restore depleted fisheries, enhanced tourism, and coastal protection of local coral reefs.	1) to develop genotyped coral nurseries, which represent the coral diversity at Cozumel Island; 2) to establish sufficient material in the coral nurseries to develop activities for education, research, technological innovation, recreation and tourism; 3) to yield sufficient material for the establishment of transplant zones; 4) to collect gametes during the spawning season for larval rearing and use the larval propagation technique to grow sexual recruits at the transplantation site	N/A	N/A	This is a collaboration between the Iberostar Group and CINVESTAV Group with the Cozumel National Natural Park and the Mexican Secretariat of Environment and Natural Resources (SEMARNAT). The program will engage with local communities, universities, government entities and tourism service providers to gather sustained funding into the future. The group is open to new partners interested in participating in the project.	N/A

The primary motivations to carry out the coral reef restoration projects are biotic and experimental to equal parts (41.7%), followed by idealistic and pragmatic reasons (both 8.3%). Biotic (36.3%) and experimental (27.3%) reasons were important secondary motivations, followed by legislative reasons (18.2%), and pragmatic/idealistic motivations (both 9.1%). All except for one of the projects reported secondary motivations. The tertiary motivations reported by 5 of the 12 projects were mainly pragmatic (80.0%) and idealistic (20.0%).

Most projects have specific objectives to optimize/scale-up restoration approaches (51.1%), followed by providing alternative, sustainable livelihood opportunities (14.9%), and then in equal parts to promote coral reef conservation stewardship and re-establish a self-sustaining, functioning reef ecosystem (12.8%). The objectives to enhance ecosystem services for the future and the reduction of population decline and ecosystem degradation accounted for only 4.2% each of the specific project objectives.

The median total cost from all projects per year is $93,000 USD (± $32,731 SE) ranging between $10,000 USD and $331,802 USD. The median spatial extent of coral reef restoration intervention is 1.0 ha (± 1.3 ha SE) ranging between 0.06 ha and 8.39 ha. Project duration was as short as 1 year and could be as long as 17 years with the median project duration of 3 years (± 1.5 years SE) to reach the project targets. Projects reported a median feasibility of 0.7 (± 0.03 SE) ranging from 0.5 to 0.8 (**Table 3**).

Table 3: Summary of total annual project costs, spatial extent of coral reef area intervened, project duration and feasibility from 12 case studies in the Spanish-speaking Caribbean and Eastern Tropical Pacific (Fundación Grupo Puntacana's restoration programs were treated as two independent projects). Error is given as standard error (± SE). Abbreviation: number of observations (N).

	Total cost per year (2018 USD)	Spatial extent (ha)	Project duration (yrs)	Feasibility (best guess)
Median	93,000<br>(± 32,731)	1.00<br>(± 1.30)	3.0<br>(± 1.5)	0.7<br>(± 0.03)
Min	10,000	0.06	1	0.5
Max	331,802	8.39	17	0.8
N	11	7	12	11

Discussion

Here we present the first comprehensive assessment of coral reef restoration projects in Spanish-speaking countries and territories of the Caribbean and Eastern Tropical Pacific (ETP), which are already being implemented or are in the initiation phase. These projects were identified through an open call for participation at the Reef Futures conference in December 2018, which aimed to bring together a large international community to develop and implement solutions to the global coral reef crisis.

We describe 12 coral reef restoration case studies in the Caribbean and Eastern Tropical Pacific that employ coral reef restoration techniques including direct transplantation, coral gardening, micro-fragmentation and larval propagation (Supplementary information, **Table S1**). With a median total project cost per year of $93,000 USD, spatial extent of 1 ha, duration of 3 years and overall project feasibility of 0.7, we show that coral reef restoration projects in these countries are more cost-effective, have overcome the barriers of scaling-up restoration interventions, are persistent through time, and have a higher likelihood of success than reported from previous literature [10, 12, 40]. For instance, the most recent published literature review on coral reef restoration presented a median value of $400,000 (2010 USD) to restore 1 ha (10,000 m^2) of coral reef, project duration of 1 year, an area intervened of 0.01 ha (108 m^2), and survival of restored corals as an item-based success indicator of 0.61 [10].

The objectives for coral reef restoration are often undocumented in the published literature, thus extracting data on the objectives from published papers may lead to skewed results. For example, Hein et al. [39] reviewed 83 published coral reef restoration studies and observed that 60% of the studies reported on evaluating the biological response of the coral reef ecosystem to transplantation (outplanting) as a main project objective. The remaining 40% of studies included the following objectives: 1) to accelerate reef recovery post-disturbance (18%), 2) to re-establish a self-sustaining, functioning reef ecosystem (48%), 3) to mitigate coral loss prior to a known disturbance (18%), and 4) to reduce population declines and ecosystem degradation (15%). In comparison, we observed that when data are elicited directly from restoration practitioners, most coral reef restoration projects in the Caribbean and Eastern Tropical Pacific had the following objectives: 1) to optimize or scale-up restoration approaches (51.1%), followed by 2) to provide alternative, sustainable livelihood opportunities (14.9%). Similarly, the projects presented here were mostly motivated by biotic reasons such as to enhance biodiversity and experimental reasons (both 41.7%), followed by idealistic/pragmatic reasons (both 8.3%). In contrast, most motivations to restore coral reefs extracted from the published literature were dominated by experimental reasons, such as to improve the restoration approach and answer ecological research questions (65.3%) [10]. Many restoration projects presented here focused on harnessing social or economic benefits from coral reef restoration such as involving the community through inclusion in activities or educational programs to raise awareness or to provide alternative, sustainable livelihood opportunities for local communities. An assessment of social, economic, and cultural benefits derived from the restoration of coral reefs has been largely ignored by the published literature, which has mostly concentrated on outcomes related to the ecology or described endeavours to improve restoration technology [10]. The present work is an attempt to bridge the gap between academics and practitioners. Academics tend to be more focused on small-experimental coral reef restoration attempts to answer questions of ecological concern, whereas practitioners are more focused on optimising and scaling-up restoration. Bridging

the gap between academics and practitioners has been identified as critical for many fields of conservation [43, 44].

Coral reef restoration in the Caribbean and Eastern Tropical Pacific face challenges similar to those of restoration efforts elsewhere in the world. For instance, the Intergovernmental Panel on Climate Change (IPCC) concluded that, if no action is taken to reduce CO_2 emissions, coral reefs would decline by 70-90 % with global warming of 1.5°C above pre-industrial levels, whereas virtually all coral reefs (> 99 percent) would be lost with 2°C warming within the next 50 years [45]. Thus, while actions to reduce CO_2 emissions are drastically needed, restoration with heat resilient species is regarded as a key strategy to rehabilitate the ecological function and ecosystem services provided by coral reefs [35]. In addition to climate change, coral reef restoration in the Caribbean and ETP face other challenges such as overfishing, sedimentation, pollution, and non-sustainable coastal development [46-51]. The recent outbreak of Scleractinian Coral Tissue Loss Disease (SCTLD) has decimated coral populations and is of major concern to those attempting to restore corals in the Caribbean. Since its onset in 2017, SCTLD has caused widespread mortality of corals, especially in the Florida Reef Tract and the Gulf of Mexico [52, 53]. The vectors causing this disease or how it can be prevented are currently unknown but are most likely bacterial [52]. A further challenge to the restoration of coral reefs in the Caribbean and ETP is the apparent lack of funding and funding strategies. None of the countries have cohesive national plans for the restoration of coral reefs similar to the Reef Restoration and Adaptation Plan in Australia which has invested AUD $100 million in 2018 to develop, trial, and deploy coral reef restoration interventions for the Great Barrier Reef (GBR) [54].

Despite the impediment of limited financial resources, considerable advances in coral reef restoration, both scaling-up of interventions and optimisation of techniques, have been achieved in Colombia, Costa Rica, Dominican Republic, Mexico and Puerto Rico. For instance, one of the largest and longest running projects (18 years) has plans to restore up to 8.4 ha, requiring outplanting 10,000 corals or up to 8,000 coral settlement bases with coral larvae per year. These interventions were led by pioneering

environmental NGOs and foundations, who often procured un-paid volunteers to carry out much of the work. The interventions were also enabled by strong partnerships initiated by the champion organization with universities (e.g. Universidad Nacional Autónoma de México, University of Puerto Rico, Universidad del Valle, Universidad Javeriana de Cali, Universidad de Costa Rica), conservation management bodies and regulators (e.g. Natural Parks administrations, Departments of Natural and Environmental Resources and the United States National Oceanic and Atmospheric Administration), associations (e.g. Fishers Association, Caribbean Hotel and Tourism Association), national and international business partners (e.g. SECORE International), international environmental NGOs (e.g. Conservation International, The Nature Conservancy), tourist service providers (e.g. the Iberostar Group), private donations (e.g. Global Giving), international grant schemes (e.g. from Deutsche Gesellschaft für Internationale Zusammenarbeit, Counterpart International, InterAmerican Development Bank (IDB)) and in large part with local community groups. Coral reef restoration still remains an underfunded area in the Spanish-speaking countries and territories of the Caribbean and ETP despite the ecosystem services restored coral reefs could provide for the regions such as food, tourism income, protection against storms and wave surges [55, 56], and reduction in insurance premiums by offering coastal protection [57].

There are a few caveats that need to be considered when assessing the data within the present work. First, this review does not contain an exhaustive list of interventions in the Spanish-speaking countries and territories of the Caribbean and ETP. Additional projects exist or are planned, but were not aware of, or chose to not participate in our open call. Second, the projects presented here varied in their specific objectives, best practice protocols, and monitoring, which hindered their comparison. For example, some projects were designed to improve and optimise the restoration approach (experimental projects), while others were more operational, i.e., aimed to scale-up the restoration of coral reefs by using already established restoration techniques. Furthermore, the projects used different best practice protocols or key indicators of restoration success, such as size of transplant and density of transplants which made a direct comparison between the projects difficult. Some projects

lacked monitoring milestones to evaluate the survival, cover and health conditions of outplanted corals beyond year one. Yet, post-restoration monitoring is an imperative method needed to confirm that outplanted corals are self-sustaining which, from an evolutionary perspective, is the ultimate goal of any restoration effort [3-5]. Third, evaluation of the overall project feasibility or the likelihood of success to reach specific project objectives is naturally linked to local conditions and circumstances, thus may be a subjective measure directly related to the experience of the practitioner. More quantitative measures of overall project feasibility (e.g., based on measurements) would be a considerable improvement over the qualitative (derived from expert elicitation) approach.

Prior to any conservation action, a prioritisation of interventions based on decision-support frameworks is recommended to help practitioners increase their planning rigor, project accountability, stakeholder participation, transparency in decisions, and learning [58]. Cost-effectiveness analysis is such a tool that allows for the evaluation and prioritisation of conservation interventions [59]. This analysis relates the costs of a project to its key outcomes or benefits i.e., the specific measures of project effectiveness [59, 60]. Although this work includes all data required for a cost-effectiveness analysis (see Supplementary material), we considered that comparing the different projects against each other will be inappropriate given the variety of their project objectives (e.g. experimental vs. operational) and the lack of standardisation in reporting on cost, feasibility and key outcomes.

Future collaborations between academics, local communities and practitioners will be crucial if we want to achieve restoration at meaningful ecological, spatial and social scales [61]. Unfortunately, the language barrier often inhibits such collaborations. For instance, Amano et al. [62] argues that languages are still a major barrier to global science by showing that more than 35% of the knowledge in conservation is missed by those who only look at peer-reviewed literature in English. Many practitioners who carry out large-scale coral restoration projects only convey their knowledge in the form of unpublished reports and grey literature [10], which adds another level of complexity to the

loss of information on restoration efforts. Here we close this gap by accessing this knowledge and overcoming the language barrier.

Conclusions

Although not previously highlighted by the published literature, there are many coral reef restoration projects currently in progress in the Spanish-speaking countries and territories of the Caribbean and Eastern Tropical Pacific. Most of these projects are being carried out by pioneering civil organizations often in strong partnerships with universities, conservation management bodies and regulators, tourism operators, the private sector, associations, and local community groups. While coral reef restoration has been portrayed as too expensive and challenging with regards to spatial scale, duration, and success, the projects presented here have shown that many of these barriers have already been overcome. These pioneering endeavours were often possible by in-kind commitments of staff and volunteers as well as involvement of the local community and tourism operators, thus socio-economic aspects play a substantial role in coral reef restoration in the Caribbean and Eastern Tropical Pacific. Strong national plans for restoration in conjunction with national and international funding are needed to multiply the already existing activities made by Latin-American organisations to improve the health and status of coral reefs in the Caribbean and Eastern Tropical Pacific. From this compilation of data and knowledge, it is apparent that it would be beneficial for coral reef restoration practitioners in this area to coordinate their efforts with each other and make sure they are sharing and implementing their best practices protocols to standardise efforts and track restoration progress by specific, measurable, achievable and repeatable metrics of success through time.

Acknowledgements

We would like to thank Nufar Charuvi, the pioneer driving the Alianza Coralina Taganga project, who, although no longer with us, continues to inspire by his example, dedication, and passion he served over the last decade for Colombian coral reefs and the local community. This manuscript has been developed upon in-kind time of the authors and has not received any financial support. We acknowledge the Iberostar Group for covering the open access publication fees of this manuscript.

References

1. SER. The SER primer on ecological restoration. Tucson, Arizona, USA: Group SPW; 2004.

2. Perrow M, Davy A. Handbook of ecological restoration. Cambridge, UK: Cambridge University Press; 2002.

3. McDonald T, Gann G, Jonson J, Dixon K. International standards for the practice of ecological restoration – including principles and key concepts.2016.

4. Edwards A. Reef rehabilitation manual. St Lucia, Australia: Coral Reef Targeted Research & Capacity Building for Management Program; 2010.

5. Baums IB, Baker AC, Davies SW, Grottoli AG, Kenkel CD, Kitchen SA, et al. Considerations for maximizing the adaptive potential of restored coral populations in the western Atlantic. Ecol Appl. 2019;0(0):e01978. doi: 10.1002/eap.1978.

6. Jeremy BCJ. Pleistocene Perspectives on Coral Reef Community Structure. American Zoologist. 1992;32(6):719-31.

7. IUCN. The IUCN Red List of Threatened Species 2019 [Downloaded on 10 January 2019]. Available from: http://www.iucnredlist.org.

8. Aronson RB, Precht WF. White-band disease and the changing face of Caribbean coral reefs. Hydrobiologia. 2001;460:25-38. doi: 10.1023/a:1013103928980. PubMed PMID: WOS:000173249100003.

9. Rodriguez-Martinez RE, Banaszak AT, McField MD, Beltran-Torres AU, Alvarez-Filip L. Assessment of Acropora palmata in the Mesoamerican Reef System. PLoS One. 2014;9(4):7. doi: 10.1371/journal.pone.0096140. PubMed PMID: WOS:000335505000064.

10. Bayraktarov E, Stewart-Sinclair PJ, Brisbane S, Bostrom-Einarsson L, Saunders MI, Lovelock CE, et al. Motivations, success, and cost of coral reef restoration. Restor Ecol. 2019;27(5):981-91. doi: 10.1111/rec.12977. PubMed PMID: WOS:000484997000012.

11. Barton JA, Willis BL, Hutson KS. Coral propagation: a review of techniques for ornamental trade and reef restoration. Rev Aquac. 2017;9(3):238-56. doi: 10.1111/raq.12135. PubMed PMID: WOS:000411513000003.

12. Boström-Einarsson L, Ceccarelli D, Babcock RC, Bayraktarov E, Cook N, Harrison P, et al. Coral restoration in a changing world - A global synthesis of methods and techniques - A report for the Reef Restoration and Adaptation Program, Subproject 1a - Review of existing technologies/pilots and new initiatives. Townsville: James Cook University, 2018.

13. Hernández-Delgado E, Mercado-Molina A, Suleimán-Ramos S. Multi-disciplinary lessons learned from low-tech coral farming and reef rehabilitation practices. I. Best management practices. In: Duque-Beltrán C, Tello-Camacho E, editors. Corals in a Changing World: InTech Publ; 2018. p. 213-43.

14. Rinkevich B. The Active Reef Restoration Toolbox is a Vehicle for Coral Resilience and Adaptation in a Changing World. Journal of Marine Science and Engineering. 2019;7(7):18. doi: 10.3390/jmse7070201. PubMed PMID: WOS:000478581900009.

15. Edwards AJ, Clark S. Coral transplantation: A useful management tool or misguided meddling? Mar Pollut Bull. 1998;37(8-12):474-87. PubMed PMID: WOS:000082454300013.

16. Precht W. Coral Reef Restoration: An Overview. Coral Reef Restoration Handbook: CRC Press; 2006. p. 55-76.

17. Rinkevich B. Conservation of coral reefs through active restoration measures: Recent approaches and last decade progress. Environ Sci Technol. 2005;39(12):4333-42. doi: 10.1021/es0482583. PubMed PMID: WOS:000229724100007.

18. Rinkevich B. Restoration strategies for coral reefs damaged by recreational activities: The use of sexual and asexual recruits. Restor Ecol. 1995;3(4):241-51. doi: 10.1111/j.1526-100X.1995.tb00091.x. PubMed PMID: WOS:A1995TL38900003.

19. Rinkevich B. Steps towards the evaluation of coral reef restoration by using small branch fragments. Mar Biol. 2000;136(5):807-12. doi: 10.1007/s002270000293. PubMed PMID: WOS:000088186100006.

20. Johnson M, Lustic C, Bartels E, Baums I, Gilliam D, Larson L, et al. Caribbean Acropora restoration guide: best practices for propagation and population enhancement. Arlington, VA: The Nature Conservancy; 2011.

21. Frias-Torres S, Goehlich H, Reveret C, Montoya-Maya PH. Reef fishes recruited at midwater coral nurseries consume biofouling and reduce cleaning time in Seychelles, Indian Ocean. Afr J Mar Sci. 2015;37(3):421-6. doi: 10.2989/1814232x.2015.1078259. PubMed PMID: WOS:000363342200014.

22. Bowden-Kerby A. Low-tech coral reef restoration methods modeled after natural fragmentation processes. Bull Mar Sci. 2001;69(2):915-31. PubMed PMID: WOS:000172609000051.

23. Shaish L, Levy G, Gomez E, Rinkevich B. Fixed and suspended coral nurseries in the Philippines: Establishing the first step in the "gardening concept" of reef restoration. J Exp Mar Biol Ecol. 2008;358(1):86-97. doi: 10.1016/j.jembe.2008.01.024. PubMed PMID: WOS:000255448300010.

24. Ferse SCA, Kunzmann A. Effects of concrete-bamboo cages on coral fragments: Evaluation of a low-tech method used in artisanal ocean-based coral farming. Journal of Applied Aquaculture. 2009;21(1):31-49. doi: 10.1080/10454430802694538.

25. Mbije NE, Spanier E, Rinkevich B. A first endeavour in restoring denuded, post-bleached reefs in Tanzania. Estuar Coast Shelf Sci. 2013;128:41-51. doi: DOI 10.1016/j.ecss.2013.04.021. PubMed PMID: WOS:000321414100005.

26. Nedimyer K, Gaines K, Roach S. Coral tree nursery©: an innovative approach to growing corals in an ocean-based field nursery. AACL Bioflux. 2011;4(4):442-6.

27. dela Cruz DW, Rinkevich B, Gomez ED, Yap HT. Assessing an abridged nursery phase for slow growing corals used in coral restoration. Ecol Eng. 2015;84:408-15. doi: 10.1016/j.ecoleng.2015.09.042. PubMed PMID: WOS:000363948000051.

28. Ng CSL, Lim SC, Ong JY, Teo LMS, Chou LM, Chua KE, et al. Enhancing the biodiversity of coastal defence structures: transplantation of nursery-reared reef biota onto intertidal seawalls. Ecol Eng. 2015;82:480-6. doi: http://dx.doi.org/10.1016/j.ecoleng.2015.05.016.

29. Forsman ZH, Page CA, Toonen RJ, Vaughan D. Growing coral larger and faster: micro-colony-fusion as a strategy for accelerating coral cover. Peerj. 2015;3:16. doi: 10.7717/peerj.1313. PubMed PMID: WOS:000364330400004.

30. Page CA, Muller EM, Vaughan DE. Microfragmenting for the successful restoration of slow growing massive corals. Ecol Eng. 2018;123:86-94. doi: 10.1016/j.ecoleng.2018.08.017. PubMed PMID: WOS:000446253500009.

31. Chamberland VF, Petersen D, Guest JR, Petersen U, Brittsan M, Vermeij MJA. New Seeding Approach Reduces Costs and Time to Outplant Sexually Propagated Corals for Reef Restoration. Sci Rep. 2017;7:12. doi: 10.1038/s41598-017-17555-z. PubMed PMID: WOS:000418644100035.

32. Banaszak A, Schutter M, Guendulain García S, Mendoza Quiroz S, Gómez Campo K. Guía práctica para la restauración con base en la producción de reclutas sexuales de corales con énfasis en Acropora palmata. México: 2018.

33. Calle-Trivino J, Cortes-Useche C, Sellares-Blasco RI, Arias-Gonzalez JE. Assisted fertilization of threatened Staghorn Coral to complement the restoration of nurseries in Southeastern Dominican Republic. Reg Stud Mar Sci. 2018;18:129-34. doi: 10.1016/j.rsma.2018.02.002. PubMed PMID: WOS:000427936600014.

34. dela Cruz DW, Harrison PL. Enhanced larval supply and recruitment can replenish reef corals on degraded reefs. Sci Rep. 2017;7:13. doi: 10.1038/s41598-017-14546-y. PubMed PMID: WOS:000413700200024.

35. Van Oppen MJH, Gates RD, Blackall LL, Cantin N, Chakravarti LJ, Chan WY, et al. Shifting paradigms in restoration of the world's coral reefs. Glob Change Biol. 2017;23(9):3437-48. doi: 10.1111/gcb.13647. PubMed PMID: WOS:000406812100003.

36. CRC. Coral Restoration Consortium 2019 [cited 2019 10 January 2019]. Available from: http://reefresilience.org/restoration/restoration-introduction/coral-restoration-consortium/.

37. Clewell AF, Aronson J. Motivations for the restoration of ecosystems. Conserv Biol. 2006;20(2):420-8. doi: 10.1111/j.1523-1739.2006.00340.x. PubMed PMID: WOS:000236064200020.

38. Hagger V, Dwyer J, Wilson K. What motivates ecological restoration? Restor Ecol. 2017;25(5):832-43. doi: 10.1111/rec.12503. PubMed PMID: WOS:000411686900019.

39. Hein MY, Willis BL, Beeden R, Birtles A. The need for broader ecological and socioeconomic tools to evaluate the effectiveness of coral restoration programs. Restor Ecol. 2017;25(6):873-83. doi: 10.1111/rec.12580. PubMed PMID: WOS:000414808200004.

40. Bayraktarov E, Saunders MI, Abdullah S, Mills M, Beher J, Possingham HP, et al. The cost and feasibility of marine coastal restoration. Ecol Appl. 2016;26(4):1055-74. doi: 10.1890/15-1077. PubMed PMID: WOS:000378092900008.

41. Iacona GD, Sutherland WJ, Mappin B, Adams VM, Armsworth PR, Coleshaw T, et al. Standardized reporting of the costs of management interventions for biodiversity conservation. Conserv Biol. 2018;32(5):979-88. doi: doi:10.1111/cobi.13195.

42. Ruiz-Jaen MC, Mitchell Aide T. Restoration Success: How Is It Being Measured? Restor Ecol. 2005;13(3):569-77. doi: 10.1111/j.1526-100X.2005.00072.x.

43. Sunderland T, Sunderland-Groves J, Shanley P, Campbell B. Bridging the Gap: How Can Information Access and Exchange Between Conservation Biologists and Field Practitioners be Improved for Better Conservation Outcomes? Biotropica. 2009;41(5):549-54. doi: 10.1111/j.1744-7429.2009.00557.x. PubMed PMID: WOS:000269700500005.

44. Milner-Gulland EJ, Fisher M, Browne S, Redford KH, Spencer M, Sutherland WJ. Do we need to develop a more relevant conservation literature? Oryx. 2010;44(1):1-2. doi: 10.1017/s0030605309991001. PubMed PMID: WOS:000274770200001.

45. IPCC. Global Warming of 1.5 °C. An IPCC special report on the impacts of global warming of 1.5 °C above pre-industrial levels and related global greenhouse gas emission pathways, in the context

of strengthening the global response to the threat of climate change, sustainable development, and efforts to eradicate poverty. Geneva, Switzerland: 2018.

46. Cortés J. Eastern Tropical Pacific Coral Reefs. In: Hopley D, editor. Encyclopedia of Modern Coral Reefs: Structure, Form and Process. Dordrecht: Springer Netherlands; 2011. p. 351-8.

47. Hubbard D. Eastern Caribbean Coral Reefs. In: Hopley D, editor. Encyclopedia of Modern Coral Reefs: Structure, Form and Process. Dordrecht: Springer Netherlands; 2011. p. 338-48.

48. Rogers CS. RESPONSES OF CORAL REEFS AND REEF ORGANISMS TO SEDIMENTATION. Mar Ecol-Prog Ser. 1990;62(1-2):185-202. doi: 10.3354/meps062185. PubMed PMID: WOS:A1990CZ63900020.

49. Cloern JE. Our evolving conceptual model of the coastal eutrophication problem. Mar Ecol-Prog Ser. 2001;210:223-53. doi: 10.3354/meps210223. PubMed PMID: WOS:000167175000019.

50. Hernandez-Delgado EA. The emerging threats of climate change on tropical coastal ecosystem services, public health, local economies and livelihood sustainability of small islands: Cumulative impacts and synergies. Mar Pollut Bull. 2015;101(1):5-28. doi: 10.1016/j.marpolbul.2015.09.018. PubMed PMID: WOS:000367630700015.

51. Jackson J, Donovan M, Cramer K, Lam VV. Status and Trends of Caribbean Coral Reefs: 1970-2012. Gland, Switzerland: 2014.

52. Meyer JL, Castellanos-Gell J, Aeby GS, Häse CC, Ushijima B, Paul VJ. Microbial Community Shifts Associated With the Ongoing Stony Coral Tissue Loss Disease Outbreak on the Florida Reef Tract. Frontiers in Microbiology. 2019;10(2244). doi: 10.3389/fmicb.2019.02244.

53. AGRRA. Coral disease outbreak: Atlantic and Gulf Rapid Reef Assessment; 2019 [cited 2019 13/10/2019]. Available from: https://www.agrra.org/coral-disease-outbreak/.

54. RRAP. Reef Restoration and Adaptation Program 2019 [cited 2019 13/10/2019]. Available from: https://www.gbrrestoration.org/the-program.

55. Woodhead AJ, Hicks CC, Norström AV, Williams GJ, Graham NAJ. Coral reef ecosystem services in the Anthropocene. Functional Ecology. 2019;33(6):1023-34. doi: 10.1111/1365-2435.13331.

56. Ferrario F, Beck MW, Storlazzi CD, Micheli F, Shepard CC, Airoldi L. The effectiveness of coral reefs for coastal hazard risk reduction and adaptation. Nat Commun. 2014;5(1):3794. doi: 10.1038/ncomms4794.

57. Beck MW, Losada IJ, Menéndez P, Reguero BG, Díaz-Simal P, Fernández F. The global flood protection savings provided by coral reefs. Nat Commun. 2018;9(1):2186. doi: 10.1038/s41467-018-04568-z.

58. Schwartz MW, Cook CN, Pressey RL, Pullin AS, Runge MC, Salafsky N, et al. Decision Support Frameworks and Tools for Conservation. Conserv Lett. 2018;11(2):e12385. doi: 10.1111/conl.12385.

59. Riegg Cellini S, Kee E. Cost-Effectiveness and Cost-Benefit Analysis. In: Newcomer K, Hatry H, Wholey J, editors. Handbook of Practical Program Evaluation2015. p. 636-72.

60. Beher J, Possingham HP, Hoobin S, Dougall C, Klein C. Prioritising catchment management projects to improve marine water quality. Environmental Science & Policy. 2016;59:35-43. doi: 10.1016/j.envsci.2016.02.005. PubMed PMID: WOS:000373548500005.

61. Gillies CL, Fitzsimons JA, Branigan S, Hale L, Hancock B, Creighton C, et al. Scaling-up marine restoration efforts in Australia. Ecological Management & Restoration. 2015;16(2):84-5. doi: 10.1111/emr.12159. PubMed PMID: WOS:000354644800002.

62. Amano T, González-Varo JP, Sutherland WJ. Languages Are Still a Major Barrier to Global Science. PLoS Biol. 2016;14(12):e2000933. doi: 10.1371/journal.pbio.2000933.

Figure captions:

Figure 1: Map of coral reef restoration projects in Spanish-speaking Latin American countries and territories.

Figure 2: Types of nurseries described in the text. a) Floating rope nurseries used in San Andrés and Providencia islands for large-scale coral gardening (Photo: Corales de Paz); b) rope nurseries by FUNDEMAR in Dominican Republic (Photo: Greg Asner); c) FUNDEMAR's floating *in situ* coral larvae rearing tank (Photo: Paul Selvaggio); d) Oceanus A.C. diver outplants nursery grown corals in Veracruz, Mexico (Photo: Oceanus A.C.); e) outplanted *Acropora palmata* coral in Puerto Morelos, Mexico (Photo: Oceanus A.C.); Raising Coral Costa Rica's tree nurseries in Costa Rica (Photo: David Garcia).

Figure 1

Click here to access/download;Figure;Fig. 1.jpg

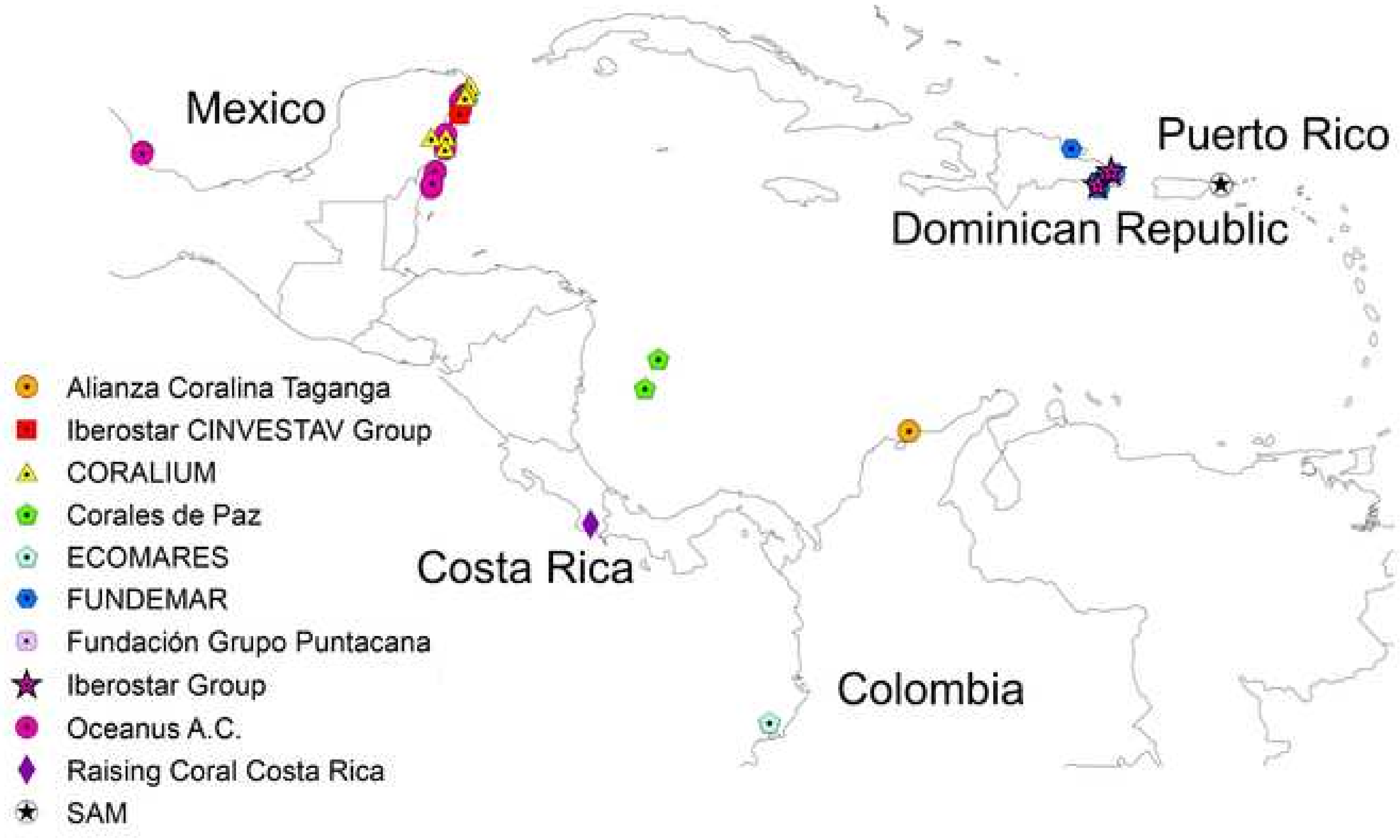

Figure 2

Click here to access/download;Figure;Fig. 2.jpg

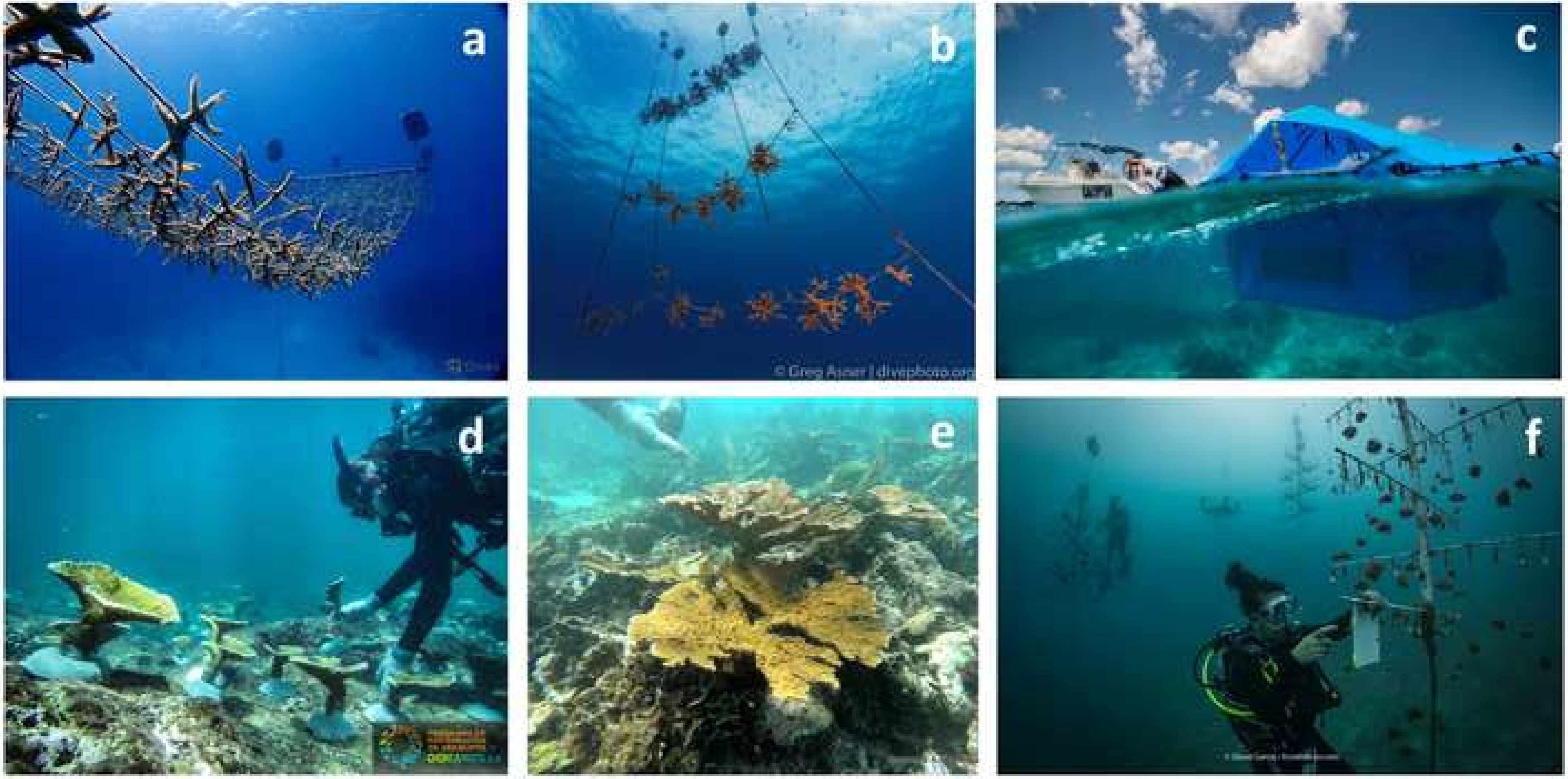

Click here to access/download
Supporting Information
Coral restoration supplementary.docx

Data

Click here to access/download
Supporting Information
Data_coral_reef_restoration.xlsx
